# Supplementary material for: A Phenomenological Model for Enthalpy Recovery in Polystyrene Using Dynamic Mechanical Spectra
Source: Polymers (Basel). 2023 Aug 29;15(17):3590. doi: 10.3390/polym15173590 (PMC10490033; doi:10.3390/polym15173590)
Supplement: Supplementary file 1 [file polymers-15-03590-s001.zip › polymers-2546675-supplementary.pdf]

Supporting Information

A Phenomenological Model for Enthalpy Recovery in Polystyrene using Dynamic Mechanical Spectra

Koh-hei Nitta*, Kota Ito, and Asae Ito

Division of Material Sciences, Graduate School of Natural Science and Technology, Kanazawa University, Kakuma Campus, Kanazawa, 920-1192, Japan

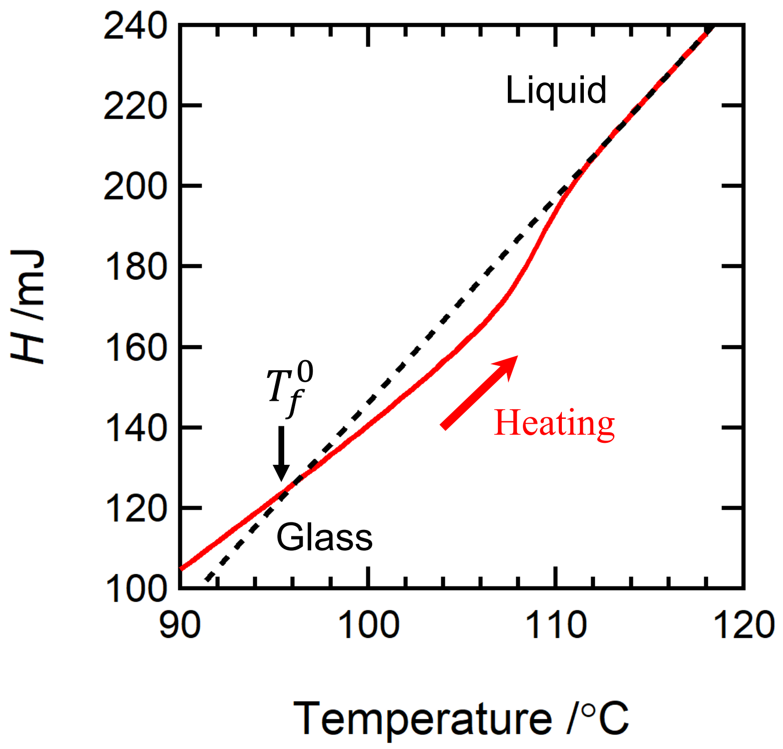

Figure S1. Enthalpy change estimated from heat flow of DSC data for PS annealed for 24h at 80 °C. The solid line corresponds to the extension (equilibrium) line of the liquid state.

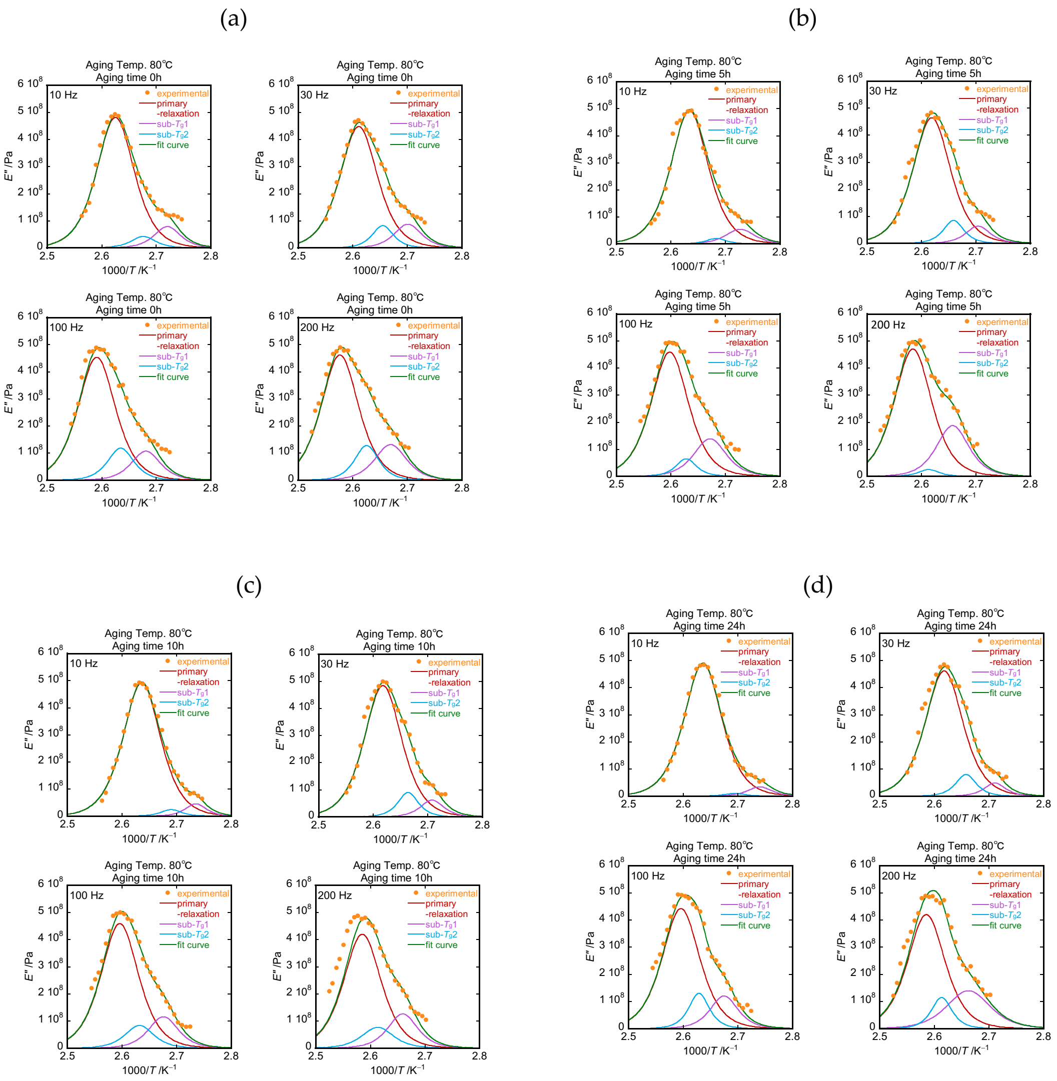

Figure S2. Curve fitting for dynamic mechanical spectra of loss modulus E'' around glass relaxation region at 1, 30, 100, and 200 Hz, for PS annealed for (a) 0, (b) 5h, (c) 10h, and (d) 24h at 80 °C.

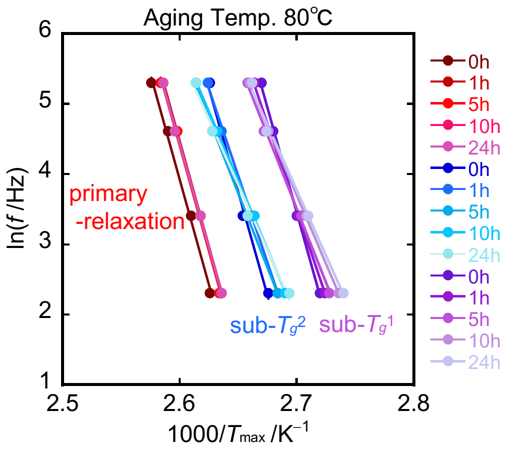

Figure S3. Arrhenius plots for primary and dual sub-glass transitions for PS annealed at 80 °C.
